# The Risk Assessment Tools for Intraoperative Hypothermia in Adults: A Scope Review

**DOI:** 10.1002/nop2.70416

**Published:** 2026-03-15

**Authors:** Hao Wang, Ping Xu, Jianying Luo, Qunhua Jiang

**Affiliations:** ^1^ Sixth People's Hospital South Campus Affiliated to Shanghai Jiaotong University Shanghai China

**Keywords:** hypothermia, intraoperative, nursing, risk assessment, tools

## Abstract

**Aims:**

To review the global scope of inadvertent intraoperative hypothermia risk assessment tools and to provide a reference for standardising risk assessment.

**Design:**

A scoping review.

**Methods:**

Studies on inadvertent intraoperative hypothermia risk assessment tools were systematically searched in Chinese and English databases and guideline websites from their inception up to December 31, 2022.

**Results:**

A total of 20 studies were included based on the inclusion and exclusion criteria. The inadvertent intraoperative hypothermia risk assessment tools were categorised into risk prediction models (*n* = 15), risk assessment tables (*n* = 4), and risk assessment systems (*n* = 1). Patient factors were reported in 20 articles, surgical factors were reported in 18 articles, anaesthetic factors were used for evaluation in 16 articles, and environmental factors were reported in 12 articles. Assessment components that were reported more than 10 times included age, body mass index, baseline body temperature, intraoperative fluid volume, surgical duration, anaesthesia duration, and room temperature.

**Impact:**

Patient and surgical factors are important components of inadvertent intraoperative hypothermia risk assessment tools. Several such tools have been developed, but their reliability and validity need evaluation through multi‐center and large‐scale clinical studies.

## 1 Introduction

Inadvertent perioperative hypothermia (IPH), defined as a perioperative decrease in body temperature below 36°C, is a common surgical complication (Song 2019; Putnamk 2015). Its incidence in foreign countries ranges from 4% to 72% and can reach as high as 90% (Vural et al. 2018). In China, the incidence of IPH is approximately 44.3% (Zhang and Zeng 2019). IPH promotes blood loss, increases blood transfusion requirements during surgery, and can lead to wound infection and cardiac complications. Additionally, IPH may prolong the recovery time and lengths of hospital and ICU stay, significantly influencing postoperative rehabilitation (National Center for Professional Quality Control in Anaesthesia 2023; Bu et al. 2019; Li et al. 2020). Therefore, early detection of IPH is important for improving the prognosis of surgical patients. Several risk assessment tools have recently been developed for inadvertent intraoperative hypothermia, but their applications vary and the main assessment components are relatively heterogeneous. Therefore, based on a review framework developed by Arksey and O'Malley (2005), we systematically analysed the main evaluation components, high‐risk critical values, and predictive effectiveness of risk assessment tools for inadvertent intraoperative hypothermia. In addition, we assessed the existing challenges in this field and provided references for standardised risk assessment of inadvertent intraoperative hypothermia.

## 2 Background

Conventional operating room systems typically do not monitor core body temperature as a routine vital sign. Artificial intraoperative monitoring of patients' core body temperature is associated with several problems, including neglect and temperature monitoring difficulties. Since core body temperature cannot be monitored continuously, it is important to predict the risk of hypothermia in a timely manner (National Center for Professional Quality Control in Anaesthesia 2023). Currently, the risk prediction tools for inadvertent intraoperative hypothermia include hypothermia risk prediction model, hypothermia risk assessment scale, and risk assessment systems. Studies of hypothermia risk factors have combined various scientific research and statistical analysis methods to predict the occurrence of hypothermia in surgical patients, resulting in statistical prediction models or scales. Most of the studies on hypothermia in surgical patients have investigated active heating measures, passive insulation measures, and comprehensive insulation nursing, and guidelines have been formulated for its prevention and treatment in surgical patients (National Collaborating Centre for Nursing and Supportive Care, Clinical Practice Guideline 2008; Hooper et al. 2010; Torossian et al. 2015). However, there are few studies on risk prediction tools for inadvertent intraoperative hypothermia in surgical patients, with most of the existing risk prediction tools based on a single research and statistical analysis method. The current hypothermia prediction tools have several shortcomings, including unclear risk degree classification, incomplete evaluation indicators for predictive performance, and lack of clinical prospective validation studies (Mendonga et al. 2021; Severens et al. 2007). This study utilised domestic and foreign literature to summarise and analyse hypothermia risk prediction tools and inadvertent perioperative hypothermia risk factors. It also discusses future research prospects for hypothermia prediction tools based on hypothermia risk factors.

### 2.1 Aims

This scoping review, based on domestic and foreign literature, analyzed the risk factors and prediction tools for inadvertent perioperative hypothermia to guide future hypothermia prediction models.

## 3 Study Design

This scoping review aimed to rapidly map the key concepts of the research area, along with the primary data sources and types of evidence available (Arksey and O'Malley 2005). We used the Joanna Briggs Institute (JBI) Reviewer's Manual methodology (Peters et al. 2020) to conduct the review, which involved the following stages: (a) identifying the review questions, (b) inclusion and exclusion criteria, (c) search strategy, (d) evidence screening and selection, (e) data extraction, (f) data analysis, and (g) presentation of results. The protocol was not registered, and the review was reported according to PRISMA Extension for Scoping Reviews.

### 3.1 Identifying the Review Questions and Eligibility Criteria

This scoping review addressed the following review questions:

1. What are the existing risk assessment tools for inadvertent intraoperative hypothermia in adults?
2. How can the risk assessment tools be applied for inadvertent intraoperative hypothermia in adults, and what are their application scenarios and development status?

The exclusion criteria were as follows:

1. Studies in languages other than Chinese or English;
2. Articles without the full text available:
3. Literature reviews and case reports;
4. Studies utilising translated or sinicized scales, and those lacking the components or methodology for the assessment tool.

### 3.2 Search Strategy

PubMed, Cochrane Library, CINAHL, Web of Science Core Collection, Embase, CNKI, and Wanfang databases were searched for relevant articles. The search also encompassed guideline websites, including the US National Guidelines Library, the UK's National Institute for Health and Clinical Excellence, and Yimai Tong. The search period extended from the date of database inception up to December 31, 2022. The English language search terms were (“hypothermia”) AND (“surgery” OR “postoperative” OR “intraoperative” OR “postoperative”) AND (“modelled” OR “predicted” OR “prognostic” OR “risk” OR “early warning” OR “predict”).

### 3.3 Study Selection

The studies were reviewed by two researchers. Initially, the bibliography was imported into the NoteExpress software to remove duplicate articles. Then, irrelevant studies were excluded based on a review of the titles and abstracts according to the inclusion and exclusion criteria. This was followed by a full text review to select the relevant articles. A third researcher was involved in case of any disagreement during the selection process to reach a consensus.

### 3.4 Data Extraction, Analysis, and Presentation of Results

Based on the JBI model, a data extraction form was developed to analyse the scope and record the characteristics of each study. The modified extraction form, based on the methodology for JBI's scoping review recommendations (Peters et al. 2020), guided data extraction. Two reviewers performed data extraction, which was independently confirmed by the third reviewer. The extracted information included authors, tool type, research methodology, main evaluation component and high‐risk threshold, reliability and validity or prediction efficiency‐related indicators, and statistical analyses.

## 4 Results

### 4.1 Study Selection

**FIGURE 1:**
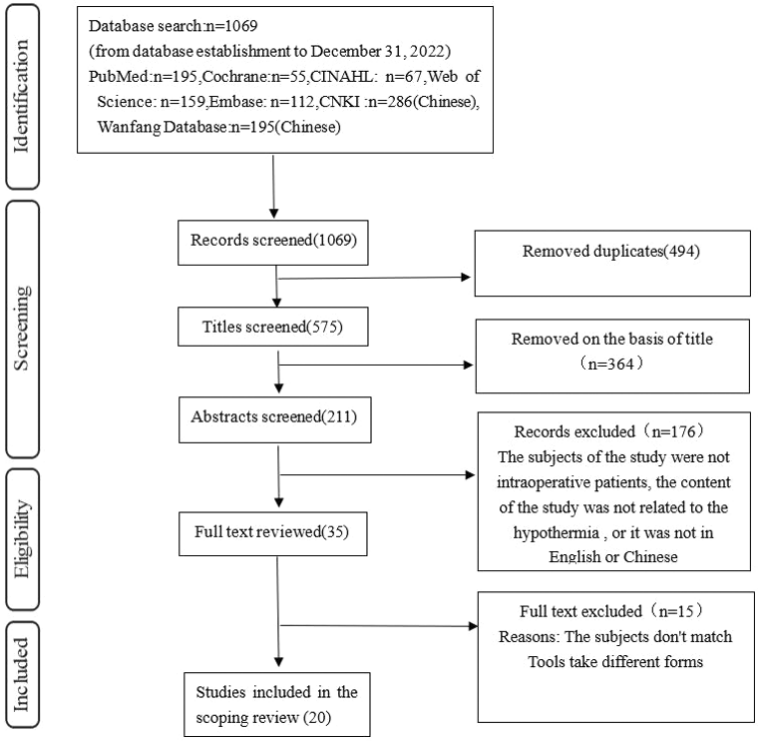
PRISMA flow diagram.

The preliminary search retrieved 1069 articles. Among these, 20 full‐text articles were selected after removing duplicates, articles that did not meet the inclusion and exclusion criteria, articles without full text, conference abstracts, and articles not published in Chinese or English language. The 20 selected articles were included after screening their titles, abstracts, and full texts in accordance with the inclusion and exclusion criteria, as shown in the PRISMA flow diagram (Figure 1).

### 4.2 Study Characteristics

The selected articles included 17 studies conducted in China, one in Japan, one in Colombia, and one in France. The research methodologies included cohort studies (Rincon et al. 2008; Desgranges et al. 2017; Pu et al. 2019; Wu et al. 2019; Chen 2020; Kong, Zhu, and Pan 2021; Kong, Deng, et al. 2021; Zhao et al. 2021; Shi et al. 2021; Li 2021a; Li 2022; Yi et al. 2017), case–control studies (Kasai et al. 2002; Liu 2022), artificial intelligence techniques (Xiang 2022), cross‐sectional surveys (Huang 2022), and the Delphi method (Yu 2020; Ke 2022; Li 2021b; Huang and Hu 2016). The detailed results are shown in Table 1.

**TABLE 1:** Characteristics of the included studies.

Author	Year	Nation	Research object	Sample size	Design
Kasai et al.	2002	Japan	Open surgery patients	A: 400 B: 50	Case–control study
Rincon et al.	2008	Colombia	Non‐cardiac surgery patients	A: 200 B: 64	Cohort study
Desgranges et al.	2017	France	Caesarean section patient	A: 359 B: −	Cohort study
Pu et al.	2019	China	Laparoscopic patients	A: 264 B: 132	Cohort study
Wu et al.	2020	China	Shoulder arthroscopic surgery patients	A: 174 B: 87	Cohort study
Chen et al.	2020	China	Non‐cardiac surgery patients	A: 2683 B: −	Cohort study
Kong et al.	2021	China	Caesarean section patient	A: 369 B: 64	Cohort study
Zhao et al.	2021	China	Patients undergoing laparoscopic surgery for endometrial cancer	A: 168 B: −	Cohort study
Shi et al.	2021	China	Patients undergoing general anaesthesia (department/disease type not specified)	A: 9155 B: 9155	Cohort study
Li	2021a	China	Urological endoscopic surgery patients	A: 342 B: 139	Cohort study
Kong et al.	2021	China	Non‐cardiac surgery patients	A: 240B: —	Cohort study
Li	2022	China	Laparoscopic patients	A: 1043 B: 313	Cohort study
Liu	2022	China	Rectal cancer surgery patient	A: 275 B: 60	Case–control study
Huang	2022	China	Neurosurgical patients	A: 664 B: 120	Cross‐sectional survey
Xiang	2022	China	Non‐cardiac surgery patients	A: 19068 B: 2157	Artificial intelligence techniques
Yu	2020	China	Non‐cardiac surgery patients	A: 120 B: −	Delphi method
Yi et al.	2017	China	Surgical patients (General surgery, liver biliary surgery, vascular surgery, etc.)	A: 3132 B: 830	Cohort study
Ke	2022	China	Non‐cardiac surgery patients	A: 150 B: −	Delphi method
Li	2021b	China	Non‐cardiac surgery patients	A: 888 B: −	Delphi method
Huang & Hu	2016	China	Non‐cardiac surgery patients	A: − B: −	Delphi method

*Note:* A is for modelling; B is for model inspection; − indicates no report.

### 4.3 Evaluation Components and Reliability and Validity Indicators of Intraoperative Hypothermia Risk Assessment Tools

The tools used for intraoperative hypothermia risk assessment were categorised into risk prediction models (*n* = 15), risk assessment tables (*n* = 4), and risk assessment systems (*n* = 1). Patient factors were reported in 20 studies, surgical factors were reported in 18 studies, anaesthetic factors were used for evaluation in 16 studies, and environmental factors were reported in 12 studies. The risk assessment tools included logistic regression analysis (*n* = 14), literature review combined with expert consultation and analytic hierarchy process (*n* = 4), random forest models (*n* = 1), and artificial intelligence deep learning modelling (*n* = 1). The components and reliability indicators for the intraoperative hypothermia risk assessment tools are presented in Table 2.

**TABLE 2:** Evaluation components and reliability and validity indices of inadvertent intraoperative hypothermia risk assessment tools.

Developer	Tool type	Main assessment component				High risk threshold	Reliability and validity	Predictive efficacy (AUC/Brier score/Youden Index/Se/Sp/accuracy rate)
		Patient factors	Surgical factors	Anaesthetic factors	Environmental factors			
Kasai et al.	Prediction model	Age, height, body mass, preoperative systolic blood pressure, preoperative heart rate						A: Se = 0.815, Sp = 0.830 B: IPH occurred when the risk rate was > 0.7; normal body temperature was maintained when the risk rate was ≤ 0.3
Rincon et al.	Prediction model	Age, body mass, baseline body temperature	Duration of surgery		Room temperature in operating room			A: AUC = 0.83, Se = 0.840, Sp = 0.730 B: AUC = 0.82, Se = 0.840, Sp = 0.670
Desgranges et al.	Prediction model	Baseline temperature, obesity	Fluid volume, core temperature at skin incision, oxytocin use		Air heating			A: AUC = 0.851 B: −
Pu et al.	Prediction model	Basal body temperature	Type of surgery	Duration of anaesthesia	Room temperature in operating room			A: AUC = 0.791, Youden index = 0.867, Se = 60.0%, Sp = 86.7% B: Se = 87.27%, Sp = 74.02%
Wu et al.	Prediction model	Basal body temperature, BMI		Duration of anaesthesia				A: AUC = O.858, Youden index = 0.603, Se = 73.5%, Sp = 86.8% B: Se = 75.68%, Sp = 92%
Chen et al.	Prediction model	Age ≥ 60 years, BMI	Amount of fluids	American Society of Anesthesiologists (ASA) grades				A: AUC = 0.683, Se = 67.1%, Sp = 60.3% B: −
Kong et al.	Prediction model	BMI, subclinical hypothyroidism during pregnancy	Intraoperative fluid loss, intraoperative irrigated abdominal fluid volume	Body temperature after anaesthesia	Duration of active insulation, operating room temperature			A: AUC = 0.888, Se = 88.0%, Sp = 74.1% B: Se = 100%, Sp = 74.0%
Zhao et al.	Prediction model	BMI	Amount of intraoperative fluid replenishment, total amount of CO_2_ injected into the abdominal cavity during the operation, and the operation time	Duration of anaesthesia				A: AUC = 0.857, Youden index = 0.729 Se = 87.3% Sp = 84.5% B: −
Shi et al.	Prediction model	Age, male BMI	Surgical position, instrumentnurse level, surgical duration	Total infusion volume, total discharge volume				A: AUC = 0.841, Youden index = 0.48 Se = 63.9%, Sp = 84.1% B: −
Li	Prediction model	BMI, basal body temperature	Surgical duration, amount of blood lost, amount of flushing fluid	Method of anaesthesia, ASA grading				A: AUC = 0.936, Youden index = 0.757 Se = 88.8%, Sp = 88.9% B: AUC = 0.914 Se = 78.13%, Sp = 89.71%
Kong et al.	Prediction model	Age, department, BMI	Duration of surgery	Duration of anaesthesia	Operating room temperature			A: AUC = 0.860, Youden index = 0.788, Se = 94.35%, Sp = 84.48% B: −
Li	Prediction model	Basal body temperature, BMI, age, sex	Duration of surgery, amount of flush fluid used intraoperatively, amount of fluid transfused intraoperatively, and surgical technique	Duration of anaesthesia, ASA classification, anaesthesia method	Operating room temperature		—	A: AUC = 0.797 Se = 78.74%, Sp = 64.0% B: −
Liu	Prediction model		Intraoperative blood loss ≥ 150 mL, intraoperative fluid replenishment > 1500 mL, total CO_2_ dosage ≥ 200 L	Duration of anaesthesia > 150 min				A: AUC = 0.797 B: AUC = 0.745
Huang	Prediction model	Age, BMI	Intraoperative fluid volume					A: AUC = 0.863, Se = 0.868 Sp = 0.764 Inside B: Se = 0.850, Sp = 0.860
Xiang	Forecasting model	Age, height, sex, BMI, basal body temperature, preoperative blood pressure, heart rate, and last preoperative haemoglobin value	Surgical duration, intraoperative urine volume, blood loss, infusion volume, total intake (including infusion and blood transfusion), surgical position	ASA score, duration of anaesthesia, total resuscitation time (time difference between entering and exiting the resuscitation room), extubation time (time between entering the resuscitation room and endotracheal intubation removal)				A: AUC = 0.724 Se = 0.516, Sp = 0.823 Inside B: prediction accuracy 74.97%
Yu	Evaluation sheet	Age, BMI, home temperature, preoperative heart rate, preoperative CA125, preoperative anxiety level, number of exercises	Surgical method, surgical duration, intraoperative blood loss, intraoperative blood transfusion, intraoperative flush fluid volume, intraoperative fluid rehydration volume, CO_2_ dosage, intraoperative fresh frozen plasma infusion	Duration of anaesthesia, method of anaesthesia, ASA classification	Operating room temperature	15.31–17.78	Cronbach's a coefficient and broken half reliability test were 0.711 and 0.777, respectively.	
Yi et al.	Rating sheet	Baseline body temperature, age, BMI, sex	Grade of surgery, amount of intraoperative fluids, amount of unheated fluids used intravenously, intraoperative irrigation, and open/endoscopic surgery	Duration of anaesthesia	Operating room temperature, insulation measures			A: AUC = 0.789 B: AUC = 0.771
Ke	Evaluation sheet	Preoperative bowel preparation	Estimated time of surgery, surgical method, skin disinfectionsite, estimated amount of cold fluid injected intravenously, estimated amount of cold fluid flushed during surgery	Method of anaesthesia, ASA level of anaesthesia	Operating room temperature, operating room cleanliness level	15	Cronbach's α coefficient = 0.780	A: AUC = 0.820 Se = 100%, Sp = 47.52 B: −
Li	Evaluation sheet	Age, BMI, basal body temperature, comorbidities	Type of surgery, estimated duration of surgery, estimated amount of intraoperative flush fluid, estimated amount of intraoperative fluids (including blood products)	Method of anaesthesia, ASA grading, and estimated duration of anaesthesia	Operating room temperature	20.5	Cronbach's α coefficient = 0.610	A: AUC = 0.748, Se = 0.707, Sp = 0.652 B: −
Huang & Hu	Evaluation system	Age, body mass, preinduction body temperature, fasting duration, metabolic disorders, psychiatric medication use	Duration of surgery, type of surgery, CO_2_ duration, intraoperative flush fluid temperature, infusion of normal temperature rehydration and stock blood, intraoperative blood loss, and surgical position	General anaesthesia	Operating room temperature			

*Note:* A is for modelling; B is for model inspection; − indicates no report.

## 5 Discussion

The main assessment components for inadvertent intraoperative hypothermia risk assessment tools included patient factors, surgical factors, anaesthetic factors, and environmental factors. All 20 of the reviewed studies focused on patient factors as the assessment component, while 18 concentrated on surgical factors, indicating a predominant use of these two types of factors in assessing inadvertent intraoperative hypothermia. The most frequently reported (> 10 times) evaluation components were age, body mass index, baseline body temperature, intraoperative fluid volume, operation time, anaesthesia time, and operating room temperature. These seven components, drawn from patient, surgical, anaesthesia, and environmental factors, were essential for establishing inadvertent intraoperative hypothermia risk assessment tools.

Anaesthetic factors were also prominently reported in 15 of the included studies. Among these factors, the ASA level (*n* = 7) and anaesthesia method (*n* = 5) were the most frequently reported. However, post‐anaesthesia temperature and other anaesthesia factors were less commonly addressed, reported only in a few studies. Further high‐quality studies are required to assess the influence of anaesthetic factors on inadvertent intraoperative hypothermia.

While several risk assessment tools for intraoperative hypothermia exist, the efficacy and validity of most of these tools remain unclear. Various studies have evaluated different tools, including risk prediction models, risk assessment tables, and risk assessment systems. The 2023 edition of operating room nursing practice guidelines does not include specific inadvertent intraoperative hypothermia risk assessment tools but discusses research methods, such as qualitative and quantitative approaches. However, the reliability of evaluation for most tools is not clearly established, and there is no widely accepted clinical risk assessment scale for inadvertent intraoperative hypothermia (Xiang 2022). Among the 20 studies included in this systematic review, 18 evaluated the predictive efficacy of the scale (Kasai et al. 2002; Rincon et al. 2008; Desgranges et al. 2017; Pu et al. 2019; Wu et al. 2019; Chen 2020; Kong, Deng, et al. 2021; Li 2022; Kong, Deng, et al. 2021; Zhao et al. 2021; Shi et al. 2021; Li 2021a, 2021b; Liu 2022; Huang 2022; Xiang 2022; Yi et al. 2017; Ke 2022), while two studies did not evaluate the predictive efficacy. Most of the evaluation tools were appraised in single‐center studies with small sample sizes. In many studies, scholars conducted external verification of some evaluation tools to ascertain their clinical significance (Kasai et al. 2002; Rincon et al. 2008; Pu et al. 2019; Wu et al. 2019; Kong, Deng, et al. 2021; Li 2021a; Liu 2022; Yi et al. 2017).

In this study, 15 prediction models were reported, with 9 developed and verified models exhibiting an area under the curve (AUC) ranging from 0.7 to 0.914 (Kasai et al. 2002; Rincon et al. 2008; Pu et al. 2019; Wu et al. 2019; Kong, Deng, et al. 2021; Li 2021a; Liu 2022; Huang 2022; Xiang 2022). However, six models were developed without evaluation based on AUC values (Desgranges et al. 2017; Chen 2020; Zhao et al. 2021; Shi et al. 2021; Kong, Deng, et al. 2021; Li 2022). Although most studies conducted initial model verification, the verification process often involved only a small sample size. It is important to conduct further multi‐center validation studies with larger sample sizes to assess the validity and stability of these models.

The majority of the included studies utilised retrospective research methods for data collection, which could introduce recall bias. Furthermore, qualitative research methods were used to outline risk assessment items, a process susceptible to subjective selectivity and influenced by the professional familiarity and cognition of relevant personnel (Yu 2020; Yi et al. 2017; Ke 2022; Li 2021b; Huang and Hu 2016). Reliability and validity are fundamental requirements for effective measurement tools and serve as basic criteria for evaluating the quality of measured data. Among the four studies reporting evaluation tables (Yu 2020; Yi et al. 2017; Ke 2022; Li 2021b), only three conducted reliability and validity evaluations (Yi et al. 2017; Ke 2022; Li 2021b), while one did not assess the reliability and validity of the tools (Yu 2020). Scholars should prioritise enhancing the evaluation of reliability and validity during the development of hypothermia assessment tables, as ensuring the reliability and validity of measurement tools is essential for obtaining reliable and accurate data. Additionally, one study constructed a risk assessment index system but did not evaluate its reliability and validity (Huang and Hu 2016).

### 5.1 Limitations

This review also had some limitations. Despite its comprehensiveness, the review was focused on peer‐reviewed English and Chinese language literature, excluding studies in other languages and grey literature. Additionally, the review included studies from various clinical settings across different countries. Consequently, the sample characteristics and sample sizes vary among the studies. Finally, we didn't conduct a strict quality assessment of included studies, and then the differences of the included literatures were not accurately explained.

## 6 Conclusions

This systematic review demonstrated the significance of patient and surgical factors in assessing hypothermia risk. While various risk assessment tools have been developed for predicting hypothermia, their clinical significance and effectiveness remain unclear. Therefore, further research and discussion are required, focusing on two key aspects: The first aspect is the integration of qualitative and quantitative research through comprehensive screening of hypothermia assessment components. Strategies should be devised to address the shortcomings observed in previous studies, such as incomplete inclusion of risk factors. Extensive data mining is essential for dynamically assessing hypothermia risk at different preoperative, intraoperative, and postoperative stages, aiming to construct a comprehensive hypothermia risk prediction model and develop an early warning system integrated into relevant information platforms. Such platforms can enhance prediction accuracy and improve clinical medical personnel's compliance in utilising risk prediction tools. The second aspect involves prospective, multi‐center studies based on big data. Most existing assessment tools stem from single‐center studies with relatively small sample sizes. External verification of these tools is important for enhancing their scientific and clinical significance. By expanding the scope of research to encompass diverse patient populations and clinical settings, the reliability and applicability of hypothermia assessment tools can be significantly improved. AUTHOR CONTRIBUTIONSHao Wang contributed to the research design, data analysis and original draft. Ping Xu and Jianying Luo conducted database searching, study selection and extracted data from studies. Qunhua Jiang participated in project administration, supervision and review. All authors approved the final version for submission.

## Author Contributions

H.W. contributed to the research design, data analysis and original draft. P.X. and J.L. conducted database searching, study selection and extracted data from studies. Q.J. participated in project administration, supervision and review. All authors approved the final version for submission.

## Funding

The authors have nothing to report.

## Disclosure

Statement: This study aimed to improve the operating room nurses' cognition of risk prediction tools for hypothermia occurrence, promote a deeper understanding of risk factors for hypothermia occurrence, provide reference for future research on hypothermia risk prediction tools, and provide a basis for dynamic continuous nursing measures perioperatively.

## Ethics Statement

The authors have nothing to report.

## Conflicts of Interest

The authors declare no conflicts of interest.

## Data Availability

All relevant data have been included in the paper.
